# Impact of prior congenital heart surgery on coronary artery bypass grafting outcomes in adults with congenital heart disease

**DOI:** 10.1007/s12055-026-02246-0

**Published:** 2026-08-03

**Authors:** Jason H. Yan, Joseph McKinnerney, Bree Cattelino, Rhian E. Davies, Rahul Kashyap

**Affiliations:** 1 Drexel University College of Medicine, 60 N. 36 Street, Philadelphia, PA 19104 USA; 2 Department of Heart and Vascular, WellSpan Health, York, PA USA; 3 Department of Research, WellSpan Health, York, PA USA

**Keywords:** Prior CHD repairs, Congenital heart disease, Adult cardiac surgery outcomes, CABG, Coronary artery bypass grafting, Adult congenital heart disease

## Abstract

**Background:**

The long-term impact of prior congenital heart disease (CHD) surgeries on adult cardiac surgery outcomes remains underexplored. Our study evaluates the influence of prior congenital heart interventions on postoperative outcomes in adult patients undergoing cardiac surgery.

**Method:**

We conducted a retrospective cohort study using the TriNetX database, analyzing adults with repaired CHD who later underwent coronary artery bypass grafting (CABG). This cohort was then compared to age-, gender-, race-, ethnicity-, comorbidity-, medication-, and CHD diagnosis-matched controls of patients with unrepaired CHD requiring CABG in adulthood. After propensity score matching, each group included 1425 individuals.

**Results:**

After matching, there were no statistically significant differences in baseline characteristics. Patients with a history of prior CHD surgical repair had significantly lower odds of 30-day all-cause mortality (odds ratio [OR] 0.33, 95% confidence interval [CI] 0.21–0.52, *p* < 0.01). However, this group also showed increased odds of hospital readmission within 30 days (OR 1.33, 95% CI 1.14–1.54, *p* < 0.01). Secondary outcomes showed higher incidence of acute kidney injury (AKI) (OR 1.42, *p* < 0.01), pleural effusion (OR 1.33, *p* < 0.01), and cardiac arrhythmias (OR 1.52, *p* < 0.01) in the CHD-repaired group. No significant differences were observed for acute myocardial infarction (MI), stroke, transient ischemic attack (TIA), or need for reoperation.

**Conclusion:**

These results suggest that patients with prior CHD repairs can successfully undergo coronary revascularization later in life despite having a distinct postoperative risk profile. However, it may also predispose patients with repaired CHD to higher rates of certain postoperative complications, including arrhythmias, pleural effusion, and AKI.

**Graphical abstract:**

**Figure:**
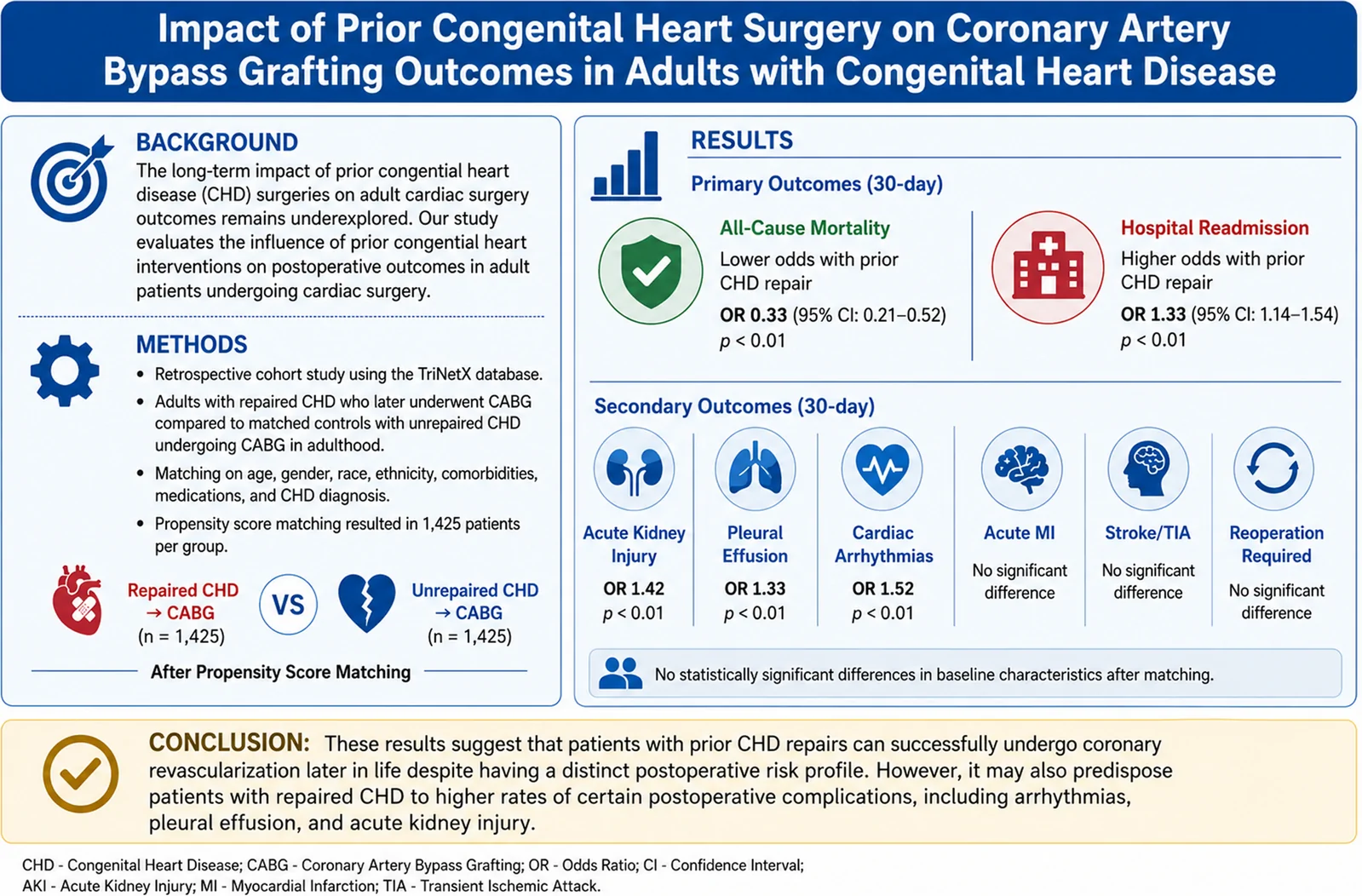

## Background

Congenital heart defects are one of the most common birth defects in neonates, with approximately 1% of live births being affected by a cardiac defect [1]. Due to advancements in surgical repair techniques for congenital heart disease (CHD), there is a rising prevalence of adults living with CHD [2]. Adults with CHD make up a substantial portion of the USA, with an estimated population size of over 1 million [3]. CHD patients are generally recommended for continued cardiology care, but are often lost to follow-up, putting them at increased risk of cardiovascular-related adverse outcomes [4]. This gap in care increases the risk for serious complications such as arrhythmias, heart failure, and coronary artery disease (CAD). This poses unique challenges for both diagnosis and management, especially if these patients undergo surgical intervention in adulthood.

Coronary artery bypass grafting (CABG) is a standard surgical treatment for significant atherosclerotic CAD and is frequently performed in older adults. Despite improvements in surgical techniques, CABG is still associated with non-negligible perioperative risks, with a 30-day mortality of up to 2.0% and 30-day morbidity of up to 14.0% [5]. CHD patients over the age of 60 have been previously identified as a population that requires higher resource utilization with an increased number of interventions and longer hospitalizations compared to their peers [6]. Additionally, CHD patients are at an overall increased risk of atherosclerosis compared to the general population [7].

CHD defects have a broad range of severity and can vary widely on the necessity of surgical intervention, with some generally warranting immediate surgical intervention while others, if small enough, may go uncorrected or resolve on their own [8]. The long-term effects of CHD, and how outcomes may differ in adulthood between those with repaired CHD versus (vs) unrepaired CHD, are poorly understood. While unrepaired CHD may appear to be clinically benign, the lingering influence it may have in adulthood, particularly regarding future CABG outcomes, remains unclear. Our study aimed to investigate the differences in perioperative and postoperative outcomes in those with repaired CHD vs unrepaired CHD undergoing CABG.

## Methods

### Study design and data source

This study was a retrospective cohort analysis utilizing de-identified electronic health record data from the TriNetX Research Network. Data for this study was obtained from the TriNetX global federated research network, which aggregates de-identified electronic health record data from participating healthcare organizations in 30 countries. TriNetX enables real-time cohort creation and analysis using standardized clinical variables. The study aimed to assess the impact of prior CHD surgical repair on outcomes in adults with CHD undergoing CABG. The data used in this study was collected on July 6, 2025, from the TriNetX Research Network, which provided access to electronic medical records (diagnoses, procedures, medications, laboratory values, genomic information) from approximately 156 million patients from 109 healthcare organizations. The study period spanned from 2005 to 2025. TriNetX, LLC was used as the data platform to conduct patient queries, extract relevant cohorts, and perform statistical comparisons across groups.

Because our study relies solely on aggregated, de-identified patient records, it was exempted from institutional review board approval and informed consent requirements. Patient data within the TriNetX Network is rigorously anonymized to comply with the de-identification standards mandated by the Health Insurance Portability and Accountability Act (HIPAA). An independent expert formally verified and updated this de-identification protocol in late 2020.

### Study population, variables, and outcomes

**Fig. 1:**
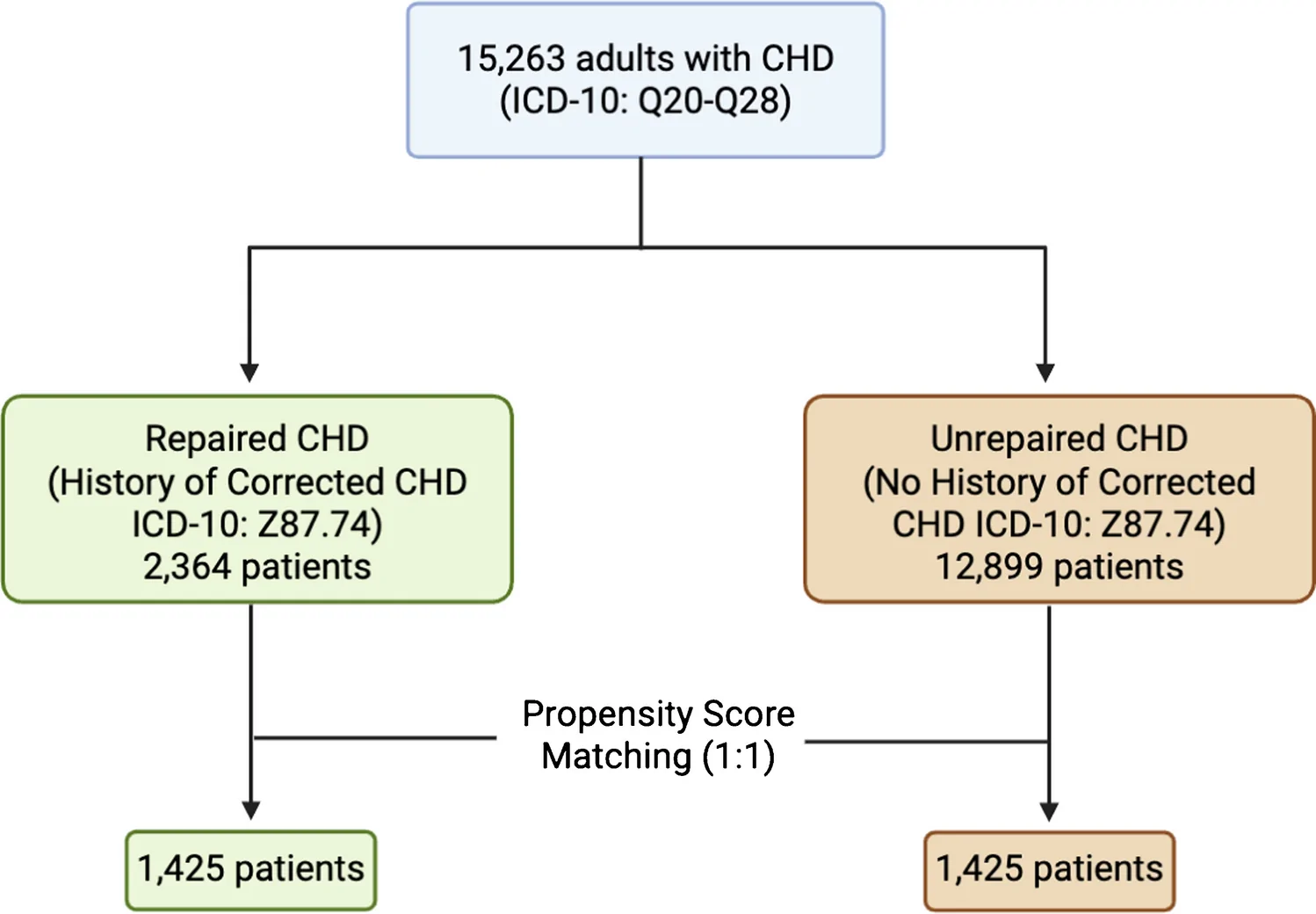
Study cohort selection and propensity score matching strategy of coronary artery bypass grafting (CABG) in adult congenital heart disease (CHD) patients

The study population included adult patients (aged 18 years or older) diagnosed with CHD, identified using International Classification of Diseases, Tenth Revision (ICD-10) codes Q20–Q26, who underwent CABG, identified by Current Procedural Terminology (CPT) codes 1006208, 1006217, and 1006200. The adult patients with a history of repaired CHD who subsequently underwent CABG were compared to a matched control group of adult patients with unrepaired CHD who also underwent CABG. The CHD surgical repair cohort was defined as patients with the CHD diagnosis and CABG mentioned above and with a personal history of (corrected) congenital malformations of the heart and circulatory system (ICD-10: Z87.74) prior to the age of 18. The control group was defined as those with the same CHD diagnosis and CABG criteria but without any personal history of (corrected) congenital malformations of the heart and circulatory system (Fig. 1).

To reduce confounding and ensure comparability between adult patients with repaired and unrepaired CHD undergoing CABG, we employed 1:1 propensity score matching using TriNetX’s built-in matching algorithm. The propensity score was derived from a logistic regression model incorporating a wide range of demographic and clinical covariates, including age at index, sex, race, and ethnicity. Clinical comorbidities included hypertension (ICD-10-CM: I10), atherosclerosis (I70), prior myocardial infarction (MI) (I21), heart failure (I50), chronic kidney disease (N18), cerebral infarction (I63), and other cerebrovascular diseases (I65). Congenital heart defect subtypes were identified using ICD-10 codes Q20–Q28 to account for variations in anatomical diagnosis. Additional variables included body mass index (BMI) (ICD-10: Z68.27–Z68.42), smoking history (F17.200), chronic pulmonary hypertension (I27.0, I27.2, I27.9), and medication use such as beta blockers, angiotensin-converting enzyme (ACE) inhibitors, calcium channel blockers, diuretics, antiarrhythmics, and lipid-lowering agents (Veterans Affairs (VA) drug classes CV100, CV800, CV200, CV700, CV300, and CV350). The presence of chronic rheumatic heart disease (I05–I09) was also included as a clinical covariate. All covariates were captured within 1 day prior to the index CABG event.

The primary outcomes assessed were 30-day all-cause mortality and hospital readmission following CABG. Hospital readmission was defined as hospital admission or emergency department visit within 30 days of CABG. Secondary outcomes were measured within 30 days of the CABG procedure as well. These included acute MI (ICD-10-CM I21), reoperation (CPT code 33530 for coronary artery bypass reoperation beyond 1 month), cerebral infarction (I63), acute kidney injury (AKI) (N17), pleural effusion (J90), transient ischemic attack (TIA) (G45.9), and arrhythmias encompassing a range of conduction disorders and abnormal rhythms (I48–I49).

All outcomes were defined using standardized diagnostic and procedural codes to ensure accuracy and reproducibility across the dataset.

### Statistical analysis

All analyses were conducted within the TriNetX platform, which uses validated algorithms and ensures compliance with HIPAA and institutional data governance standards. Post-matching, we measured the odds ratio (OR), reported with corresponding 95% confidence intervals (CI) and the associated *p*-values to determine statistical significance. A two-sided *p*-value < 0.05 was considered statistically significant.

## Results

The initial study population consisted of 15,263 adults with CHD who underwent CABG. This was further divided into two groups based on if they had undergone a CHD repair operation prior to CABG surgery. The first group comprised people who had previously had a CHD repair and contained 2364 study participants prior to matching. While the control group included those with no prior repair and had 12,899 individuals. After propensity score matching was completed through the TriNetX database’s built-in matching function, each group had 1425 individuals.

**Fig. 2:**
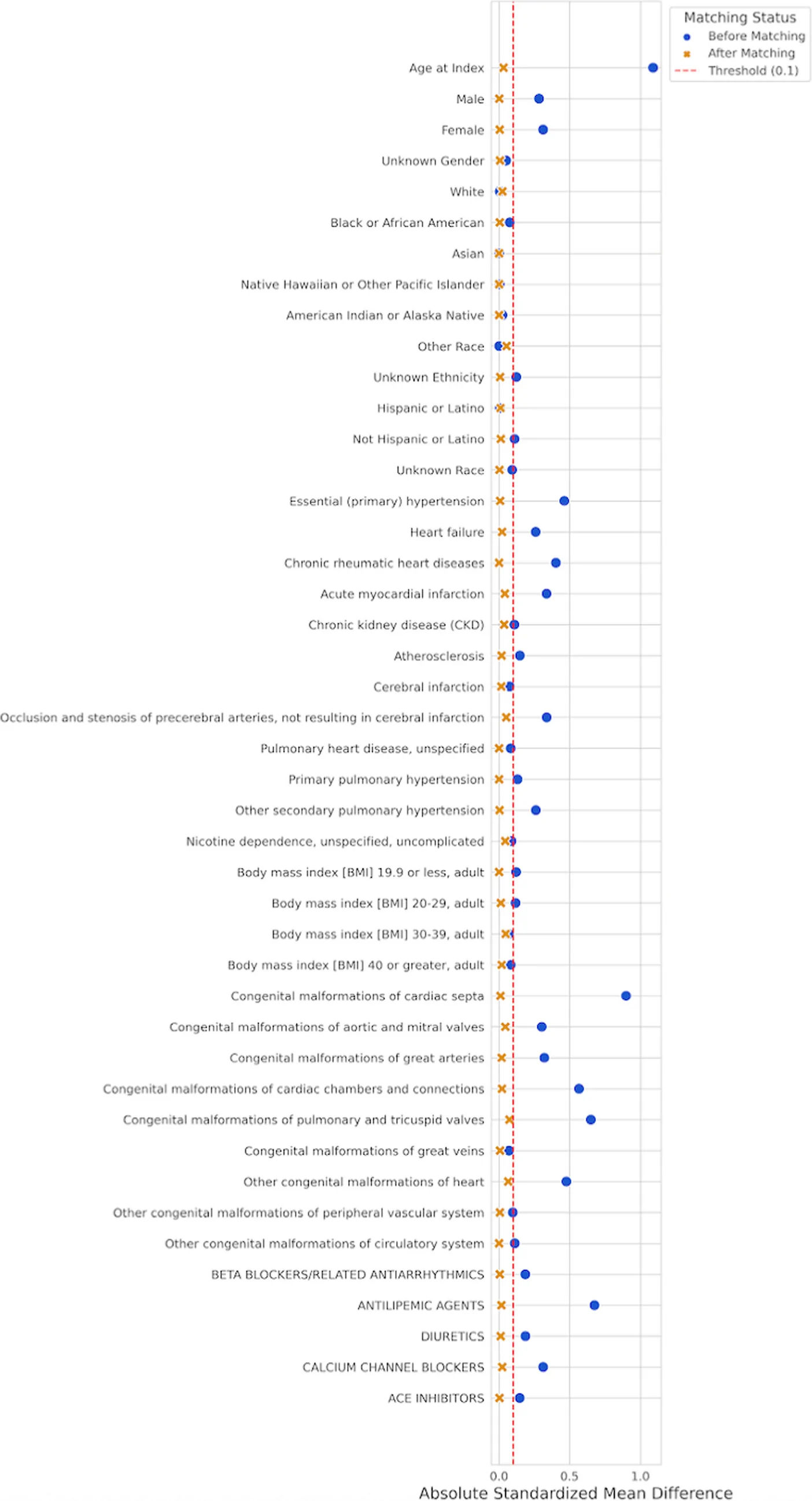
Standardized mean differences before and after propensity-score matching. Blue circles indicate values before matching, orange crosses indicate values after matching, and the dashed line indicates the prespecified threshold of 0.1. ACE, angiotensin-converting enzyme; BMI, body mass index; CKD, chronic kidney disease

Demographics between the two groups after propensity score matching were not statistically different across the baseline characteristics, as outlined in Table 1. The average age of a study participant was around 59 years old, and the majority were male (63.7%). Further balancing of the two groups was done based on common comorbidities such as hypertension or heart failure, BMI, and the individual’s medications to account for possible confounding variables. After propensity matching, there were no statistical differences between the two groups for these characteristics (Fig. 2).

**Table 1:** Baseline demographic and clinical characteristics of adult congenital heart disease (*CHD*) patients undergoing coronary artery bypass grafting (*CABG*), comparing those with prior CHD repair to those without, after propensity score matching

	CHD-repaired (*N* = 1425)	CHD-unrepaired (*N* = 1425)	*P*-value
**Demographics**			
Age at index (mean ± standard deviation)	58.95 (13.44)	58.54 (12.79)	0.40
Male	908 (63.72%)	907 (63.65%)	0.97
Female	453 (31.79%)	456 (32.00%)	0.90
Unknown gender	64 (4.49%)	62 (4.35%)	0.86
**Race**			
White	1119 (78.53%)	1105 (77.54%)	0.53
Black or African American	120 (8.42%)	122 (8.56%)	0.89
Asian	36 (2.53%)	36 (2.53%)	1.00
Native Hawaiian/Pacific Islander	10 (0.70%)	10 (0.70%)	1.00
American Indian/Alaska Native	10 (0.70%)	10 (0.70%)	1.00
Other race	31 (2.18%)	43 (3.02%)	0.16
Unknown race	102 (7.16%)	101 (7.09%)	0.94
**Ethnicity**			
Hispanic or Latino	67 (4.70%)	64 (4.49%)	0.79
Not Hispanic or Latino	1136 (79.72%)	1143 (80.21%)	0.74
Unknown ethnicity	222 (15.58%)	218 (15.30%)	0.84
**Comorbidities**			
Essential hypertension	1005 (70.53%)	1000 (70.18%)	0.84
Heart failure	773 (54.25%)	758 (53.19%)	0.57
Chronic rheumatic heart disease	552 (38.74%)	552 (38.74%)	1.00
Acute myocardial infarction	390 (27.37%)	416 (29.19%)	0.28
Chronic kidney disease	336 (23.58%)	358 (25.12%)	0.34
Atherosclerosis	275 (19.30%)	285 (20.00%)	0.64
Cerebral infarction	208 (14.60%)	216 (15.16%)	0.67
Occlusion/stenosis of precerebral arteries	276 (19.37%)	305 (21.40%)	0.18
Pulmonary heart disease	10 (0.70%)	10 (0.70%)	1.00
Primary pulmonary hypertension	39 (2.74%)	39 (2.74%)	1.00
Other secondary pulmonary hypertension	347 (24.35%)	349 (24.49%)	0.93
Nicotine dependence	219 (15.37%)	242 (16.98%)	0.24
**Body mass index (BMI)**			
BMI < 19.9	25 (1.75%)	25 (1.75%)	1.00
BMI 20–29	187 (13.12%)	181 (12.70%)	0.74
BMI 30–39	343 (24.07%)	315 (22.11%)	0.21
BMI > 40	113 (7.93%)	106 (7.44%)	0.62
**Congenital heart disease (CHD) diagnosis**			
Cardiac septal malformations	576 (40.42%)	569 (39.93%)	0.79
Aortic/mitral valve malformations	400 (28.07%)	372 (26.11%)	0.24
Great artery malformations	149 (10.46%)	157 (11.02%)	0.63
Cardiac chambers/connections malformations	60 (4.21%)	66 (4.63%)	0.58
Pulmonary/tricuspid valve malformations	16 (1.12%)	29 (2.04%)	0.05
Great vein malformations	14 (0.98%)	15 (1.05%)	0.85
Other heart malformations	216 (15.16%)	250 (17.54%)	0.09
Peripheral vascular malformations	21 (1.47%)	22 (1.54%)	0.88
Other circulatory malformations	10 (0.70%)	10 (0.70%)	1.00
**Medication use**			
Beta-blockers	1045 (73.33%)	1048 (73.54%)	0.90
Antiarrhythmics	1033 (72.49%)	1013 (71.09%)	0.41
Antilipemic agents	979 (68.70%)	968 (67.93%)	0.66
Diuretics	829 (58.18%)	821 (57.61%)	0.76
Calcium channel blockers	712 (49.97%)	728 (51.09%)	0.55
Angiotensin-Converting Enzyme inhibitors	536 (37.61%)	538 (37.75%)	0.94

Propensity score matching was also done for the different types of CHD diagnosis, so that both groups had a similar distribution. The most common form of CHD in both groups was malformation of the cardiac septa (atrial or ventral septal defect) with this making up 40% of CHD cases. Defects of the aortic and mitral valves, unclassified heart defects, and malformations of the great arteries were the second, third, and fourth most common diagnoses, respectively. Other forms of CHD were also included: defect of cardiac chambers and connections (4.21% vs 4.63%), malformations of pulmonary and tricuspid valves (1.12% vs 2.04%), unclassified defects of the peripheral vascular system (1.47% vs 1.54%), malformations of great veins (0.98% vs 1.05%), and unclassified defects of the circulatory system (0.7% in both groups).

### Outcomes

The primary outcomes measured were 30-day all-cause mortality and hospital readmission (Table 2). For the CHD-repaired group, there was a statistically significant association with a decrease in 30-day all-cause mortality (OR 0.33, 95% CI 0.21–0.52, *p* < 0.01). However, for this same group, there was also an association with an increase in hospital readmission (OR 1.33, CI 1.14–1.54, *p* < 0.01). The survival difference between groups became evident within the first week and remained consistent through day 30, supporting the finding of reduced early mortality in patients with prior CHD surgical correction. Log-rank testing confirmed a statistically significant difference in survival between the two groups (*p* < 0.01).

**Table 2:** Postoperative outcomes at 30 days following coronary artery bypass grafting (*CABG*) in adult congenital heart disease (*CHD*) patients, comparing those with prior *CHD* repair to those without, with corresponding odds ratios

Outcome	CHD-repaired *N *(%)	CHD-unrepaired *N* (%)	Odds ratio	95% confidence interval	*P*-value
All-cause mortality	25 (1.8%)	74 (5.2%)	0.33	(0.21, 0.52)	< 0.01
Acute myocardial infarction	159 (11.2%)	172 (12.1%)	0.92	(0.73, 1.15)	0.45
Hospital readmission	612 (42.9%)	516 (36.2%)	1.33	(1.14, 1.54)	< 0.01
Stroke	82 (5.8%)	68 (4.8%)	1.22	(0.88, 1.70)	0.24
Acute kidney injury	247 (17.3%)	183 (12.8%)	1.42	(1.16, 1.75)	< 0.01
Pleural effusion	533 (37.4%)	442 (31.0%)	1.33	(1.14, 1.55)	< 0.01
Transient ischemic attack	12 (0.8%)	10 (0.7%)	1.20	(0.52, 2.79)	0.67
Arrhythmia	682 (47.9%)	537 (37.7%)	1.52	(1.31, 1.76)	< 0.01

Secondary outcomes that were measured were generally mixed, showing either no significant differences between the groups or an associated increase in occurrence for the CHD repair group. For instance, AKI (OR 1.42, 95% CI 1.16–1.75, *p* < 0.01), pleural effusion (OR 1.33, CI 1.14–1.55, *p* < 0.01), and cardiac arrhythmias (OR 1.52, 95% CI 1.31–1.76, *p* < 0.01) all occurred at significantly higher rates in the CHD-repaired group, while those that did not show a significant association included acute MI (OR 0.92, CI 0.73–1.15, *p* = 0.45), reoperation (OR 1, CI 0.42–2.41, *p* = 1), stroke (OR 1.22, CI 0.87–1.7, *p* = 0.24), and TIA (OR 1.2, CI 0.52–2.8, *p* = 0.67).

## Discussion

In our study, we found that the all-cause mortality rates were lower in adult patients with previously repaired CHD, but hospital readmission rates, AKI, pleural effusion, and arrhythmias were higher. The rate of MI, stroke, TIA, or reoperation did not differ significantly between the groups.

Our study showed that adult patients with previously repaired CHD are associated with lower all-cause mortality after CABG compared to patients with unrepaired CHD. CABG is generally considered safe for adult CHD patients despite their complex cardiovascular pathophysiology [9]. A previous study demonstrated that adult CHD patients are associated with higher incidences of in-hospital mortality and postoperative complication rates compared to adults without CHD [10]. The observed survival benefit may stem from the advantages of early hemodynamic correction and the routine follow-up which are commonly provided to surgically treated CHD patients. The surgery may help to stabilize cardiovascular physiology in the long term, reducing the cumulative burden of volume or pressure overload that could have contributed to long-term mortality [11]. Patients who underwent surgical correction may have had more frequent cardiology follow-up, in line with American College of Cardiology and American Heart Association guidance for adults with corrected CHD and earlier detection of coronary disease which allowed for early intervention [12]. However, as a considerable proportion of patients in our study had relatively lower-risk congenital conditions, with atrial septal defect (ASD) and ventricular septal defect (VSD) accounting for 576 patients (40.42%) in the CHD-repaired group and 569 patients (39.93%) in the CHD-unrepaired group, with no significant difference between the groups, this observation should be interpreted cautiously, as the retrospective nature of the study and the heterogeneity of the population may introduce potential confounding factors influencing early mortality.

The repaired group experienced a lower mortality rate, but higher hospital readmission rates and higher rates of AKI, pleural effusion, and arrhythmias following CABG. While it may seem counterintuitive that the CHD-repaired group experienced lower mortality alongside higher morbidity, this could reflect the distinct clinical trajectory of these patients. Patients with a history of CHD repair are more likely to receive regular cardiology follow-up, facilitating early CAD intervention and better preoperative medical optimization. Previous studies have shown that children with CHD have increased healthcare utilization, higher rates of chronic conditions, and potential limitations in physical activity or overall well-being than those without CHD [13]. Consequently, an adult CABG for these patients is a redo procedure, which is technically more demanding due to pericardial adhesions and increased risk of complications. Patients who have undergone CHD repair can encounter anatomical and technical challenges for subsequent operations such as residual anatomical defects, physiological abnormalities, and the development of arrhythmias [14, 15]. These factors might lead to management difficulties and kidney stress during surgery and higher arrhythmogenic potential. This may contribute to greater perioperative complications and resource utilization.

The rates of MI, stroke, TIA, and reoperation were not significantly different between the repaired and unrepaired groups. Therefore, prior CHD repair status may not be an important factor in determining acute ischemic or structural cardiac complications during or after CABG. Instead, shared cardiovascular risk factors and surgical variables likely play a larger role in influencing these outcomes. A previous study showed that variables such as increasing age, known cerebrovascular disease, diabetes mellitus, and emergent operative status are predictive of stroke after major cardiac surgeries [16]. It was demonstrated that the risk of CABG can be minimized even for patients with adult CHD by optimizing standard cardiovascular care and individualizing surgical planning [17]. Previous data has shown that CABG postoperative complications such as MI are most strongly associated with arteriosclerosis and surgical factors rather than structural difference of the heart [18]. This is consistent with our study as there are no significant differences in atherosclerosis status between the CHD-repaired and CHD-non-repaired control groups after propensity score matching.

The relatively high prevalence of chronic rheumatic heart disease and pulmonary hypertension in this cohort warrants further discussion. Rheumatic valvular disease may potentially lead to increased rates of CABG through elevated lipoprotein(a) levels that drive accelerated atherosclerosis and CAD. A recent study found that serum lipoprotein(a) concentrations were significantly higher in patients with rheumatic heart valvular disease compared to controls (21 mg/dL vs 17 mg/dL, *p* < 0.001), which is associated with atherosclerotic cardiovascular diseases [19, 20]. As a result, some patients with rheumatic valvular disease may also develop CAD over time, which can necessitate CABG in adulthood. Importantly, the TriNetX database does not allow determination of whether valve repair or replacement was performed concurrently with CABG, nor does it provide data on lesion severity or hemodynamic burden. Consequently, it remains unclear whether the presence of rheumatic valvular disease or pulmonary hypertension was directly related to the indication for CABG in these cases. As such, these conditions were analyzed as comorbidities rather than primary indications for surgery.

### Strengths

Our study has several notable strengths. Our study used data from a large database that included millions of de-identified patients, which can help expand the generalizability of our findings. This approach is particularly useful for studying CHD, which remains relatively uncommon among adults. We used standardized ICD-10 and CPT coding in the study to ensure reproducible methodology and consistent diagnostic and procedural data for future research and clinical decision-making by clinicians. To account for the differences in baseline characteristics of the two inherently different patient groups, we used propensity score matching that let us match baseline characteristics between CHD-repaired and unrepaired groups to minimize confounding effects for valid outcome comparisons to the best of our abilities. Finally, to our knowledge, this study presents the first investigation comparing post-CABG outcomes in adult CHD patients who have undergone CHD correction procedures with those that have unrepaired CHD, allowing us to lay the foundation for addressing an important knowledge gap in one of the most studied cardiac procedures to date.

### Limitations

However, there are a few important limitations that we also need to recognize. Our retrospective cohort analysis faces the fundamental limitations that it depends on historical data selection bias and residual confounding. Propensity score matching helps balance the groups but does not eliminate all unmeasured variables which include surgical complexity along with functional status, socioeconomic factors, and outpatient management practices that can have a significant impact on surgical outcomes. Left ventricular ejection fraction (EF) was not included as a matching variable because EF data are inconsistently captured within the TriNetX database, and inclusion would have substantially reduced the sample size and introduced selection bias. Given the limitations of the study and the platform that we utilized, we are unable to separate patients by CHD type or severity. This serves as a major limitation since CHD patients exhibit diverse clinical characteristics. Patients with severe CHD subtypes require surgical repair but also develop additional serious long-term complications which could affect the observed relationship between repair status and adverse outcomes. Patients who received CHD repair demonstrate more complex cardiac anatomy and previous surgical modifications, leading to increased occurrences of arrhythmias, pleural effusions, and AKI.

The TriNetX database also does not provide access to operative notes, which represents a significant study limitation. We are unable to discern the specific CABG techniques that are used by cardiac surgeons as individualized surgical planning can also play a critical role in the outcome of the adult CHD patients undergoing future cardiac procedures. The surgical approaches between groups may differ through graft selection, cannulation strategy, and myocardial protection methods, thus affecting the observed perioperative complications. Furthermore, the TriNetX database does not provide information on surgeon type (adult vs congenital) or the hospital and clinical context in which operations were performed, which may introduce potential unmeasured confounding. Another inherent limitation of the analysis is the applicability of comparing patients with repaired CHD undergoing CABG to those with unrepaired CHD undergoing CABG. The former group typically undergoes a redo sternotomy, whereas the latter represents patients with unrepaired and physiologically heterogeneous CHD undergoing CABG as a primary operation, resulting in fundamental differences in operative complexity and baseline risk. However, despite the more challenging redo sternotomy, we found that patients with repaired CHD were associated with lower mortality, suggesting that further studies are required to discern this finding.

Consequently, this study should be viewed as hypothesis-generating. Our aim is to highlight a distinct postoperative risk profile in the adult CHD-repaired population that warrants further investigation. The “counterintuitive” finding of lower mortality despite higher morbidity suggests that while preoperative optimization is effective, the technical burden of redo surgery and the electrophysiological legacy of prior repairs may remain as significant hurdles. In addition, we were unable to assess critical perioperative metrics such as chest tube output, re-exploration for bleeding, or blood product transfusion requirements, as these data points were not systematically captured in our initial matched cohorts. The absence of these factors severely limits the causal interpretation of the observed mortality differences, and the lower odds of mortality in the repaired group may heavily reflect unmeasured baseline operative risk disparities. These findings provide a justification for future prospective studies that can collect the granular operative data necessary to refine individualized surgical planning. Additionally, the analysis of specific secondary outcomes is limited by the availability of postoperative data.

Additionally, our study using the TriNetX database relies heavily on accurate medical coding. The use of ICD-10 code Z87.74 to identify a history of CHD repair is susceptible to undercoding, which may have led to the misclassification of some previously repaired patients into the unrepaired cohort. Propensity score matching was conducted using TriNetX’s built-in proprietary matching algorithm; therefore, manual adjustment of caliper widths and direct visualization of convergence parameters were not possible. This may limit the exact reproducibility of the matching process.

Future research needs access to detailed operative data to determine if surgical methods vary based on CHD repair status and whether these variations affect short-term and long-term results. More prospective studies with detailed anatomical and procedural data need to be conducted to establish better risk assessment methods for individualized perioperative care.

## Conclusion

Adults who had their CHD repaired before undergoing CABG had lower 30-day all-cause mortality rates than those with unrepaired CHD. However, they also had higher rates of hospital readmission, AKI, pleural effusion, and arrhythmias. These findings suggest that early CHD repair may offer long-term survival benefits but can lead to increased perioperative complications. There were no significant differences in rates of MI, stroke, TIA, or reoperation between groups. Careful perioperative planning and long-term follow-up are essential for optimizing outcomes in this growing patient population. Future prospective studies are needed to better understand how CHD subtype and surgical history affect adult cardiac surgical outcomes. Given the limitations of retrospective database studies, these findings should be viewed as hypothesis-generating.

## Data Availability

The data supporting the findings of this study are available within the article.

## Declarations

### AI declaration

During the preparation of this work, the author(s) used Google Gemini in order to improve readability and English grammar. After using this tool/service, the author(s) reviewed and edited the content as needed and take(s) full responsibility for the content of the publication.
